# Symmetry breaking in mass-recruiting ants: extent of foraging biases depends on resource quality

**DOI:** 10.1007/s00265-016-2187-y

**Published:** 2016-07-30

**Authors:** R. I’Anson Price, C. Grüter, W. O. H Hughes, S. E. F. Evison

**Affiliations:** 1 School of Biology, University of Leeds, Leeds, UK; 2 Department of Ecology and Evolution, Biophore, University of Lausanne, 1015 Lausanne, Switzerland; 3 Institute of Zoology, Johannes Gutenberg University Mainz, Johannes von Müller Weg 6, 55099 Mainz, Germany; 4 School of Life Sciences, University of Sussex, Brighton, BN1 9QG UK; 5 Department of Animal and Plant Sciences, University of Sheffield, Sheffield, S10 2TN UK

**Keywords:** *Monomorium pharaonis*, Trail pheromones, Symmetry breaking, Colony organisation, Foraging

## Abstract

**Abstract:**

The communication involved in the foraging behaviour of social insects is integral to their success. Many ant species use trail pheromones to make decisions about where to forage. The strong positive feedback caused by the trail pheromone is thought to create a decision between two or more options. When the two options are of identical quality, this is known as symmetry breaking, and is important because it helps colonies to monopolise food sources in a competitive environment. Symmetry breaking is thought to increase with the quantity of pheromone deposited by ants, but empirical studies exploring the factors affecting symmetry breaking are limited. Here, we tested if (i) greater disparity between two food sources increased the degree to which a higher quality food source is favoured and (ii) if the quality of identical food sources would affect the degree of symmetry breaking that occurs. Using the mass-recruiting Pharaoh ant, *Monomorium pharaonis*, we carried out binary choice tests to investigate how food quality affects the choice and distribution of colony foraging decisions. We found that colonies could coordinate foraging to exploit food sources of greater quality, and a greater contrast in quality between the food sources created a stronger collective decision. Contrary to prediction, we found that symmetry breaking decreased as the quality of two identical food sources increased. We discuss how stochastic effects might lead to relatively strong differences in the amount of pheromone on alternative routes when food source quality is low.

**Significance statement:**

Pheromones used by social insects should guide a colony via positive feedback to distribute colony members at resources in the most adaptive way given the current environment. This study shows that when food resources are of equal quality, Pharaoh ant foragers distribute themselves more evenly if the two food sources are both of high quality compared to if both are of low quality. The results highlight the way in which individual ants can modulate their response to pheromone trails which may lead colonies to exploiting resources more evenly when in a resource rich environment.

**Electronic supplementary material:**

The online version of this article (doi:10.1007/s00265-016-2187-y) contains supplementary material, which is available to authorized users.

## Introduction

Several methods of communication are used by social insects to recruit nestmates to profitable resources. For example, honey bees use the waggle dance to communicate the position of a food source and its odour (von Frisch 1967; Farina et al. 2012), while ants use tandem running (Möglich and Hölldobler 1974), adult transport (Hölldobler and Wilson 1990) and pheromones (Hangartner 1970; Beckers et al. 1992; Czaczkes et al. 2015). In many ant species, trail-based pheromones are the most important means of communication due to the large size of their colonies and the fact that they are substrate bound (Hölldobler and Wilson 1990). The pheromones are deposited along foraging trails, providing positive and negative feedback to make decisions on colony foraging (Hölldobler and Wilson 1990; Traniello and Robson 1995; Camazine et al. 2001; Ratnieks 2008; Czaczkes et al. 2015). These decisions will ultimately affect fitness due to their influence on resource acquisition, leading to evolutionary pressure to make more accurate decisions (Marshall et al. 2009; Chittka et al. 2009; Stroeymeyt et al. 2010). In social insects, an individual’s decision, for example, if an individual lays an attractive pheromone trail, can be scaled to affect the fitness of the entire colony, especially in mass-recruiting species as it will influence the action of nestmates via positive feedback. Social insects are required to make these decentralised decisions during processes such as choosing a new nest site (Mallon et al. 2001; Evison et al. 2012b), avoiding predation (Lamon and Topoff 1981), and organisation while foraging for food (Beckers et al. 1992; Sumpter and Beekman 2003; Devigne and Detrain 2006).

The ability of ants to control the intensity of pheromone deposition is a well-established phenomenon that has been demonstrated empirically in multiple studies (Hölldobler et al. 1978; Beckers et al. 1992; Jackson and Châline 2007). For example, when food sources vary in quality, Pharaoh ants are known to modulate their trail laying behaviour (Jackson and Châline, 2007), which allows them to distribute their workforce to forage at a higher quality resource (Sumpter and Beekman, 2003). This food-quality-dependent modulation of communication during foraging focuses the colony’s foraging decisions and enables them to exploit high quality food sources more thoroughly. Modelling of this behaviour suggests that a colony should be expected to bias their foraging towards one feeder or the other, rather than exploit both equally, even if a resource quality is identical (Sumpter and Beekman, 2003). Indeed, in nature, it is often observed that mass-recruiting ants with access to multiple unrestricted food sources tend to concentrate their foragers on one resource, a phenomenon known as symmetry breaking (Beckers et al. 1990; Portha et al., 2002; Sumpter and Beekman 2003; Grüter et al. 2012; Jeanson et al. 2012). This phenomenon allows a rapid build-up of individuals at a single resource (Shaffer et al. 2013), which can be essential for finding and monopolising a nest site or food source rapidly (Detrain and Deneubourg 2008).

It has been argued that the degree of symmetry breaking at two identical food sources is positively related to the quality of the food sources, as food quality affects the amount of pheromone and the number of ants on the trail (Sumpter 2010). Portha et al. (2002) found that symmetry breaking was stronger when *L. niger* colonies exploited sucrose solution compared to when foraging for protein food, which has been interpreted as support for this hypothesis (Sumpter 2010). On the other hand, Weber’s law states that the perceived difference between two stimuli may depend, largely, on the proportional size of the two stimuli (Deco et al. 2007; Perna et al. 2012; Czaczkes et al. 2015). There is increasing evidence that the response of individual ants to pheromone trails follows Weber’s law (Perna et al. 2012; von Thienen et al. 2014). According to this law, in situations when pheromone deposition intensity on the trail network is generally low, such as towards low quality food sources, the decision of individual ants to deposit pheromone will have a disproportionate effect on the relative trail strength. For high quality food sources, the opposite is true; while the actual difference in the amount of pheromone might be larger, the proportional difference may be smaller because a large proportion of ants will deposit pheromone on both branches. Overcrowding at food sources could also lead to increased use of alternate branches or food sites and result in reduced symmetry breaking, which can also allow colonies to maintain an optimal rate of food return (Grüter et al. 2012). It is clear that there are multiple possible mechanisms that will lead to flexibility in exploiting food resources; however, little is known about how the quality of food affects the degree of symmetry breaking.

The Pharaoh ant, *Monomorium pharaonis*, has a sophisticated and well-studied multicomponent pheromone system (Jackson et al. 2004; Ratnieks 2008). Trails are laid using two volatile, short-lived pheromones (a positive attractant and a negative, no entry, pheromone) and a non-volatile, long-lived, attractant (positive) pheromone, which together allow the Pharaoh ant to efficiently exploit ephemeral food sources and migrate nests very quickly (Robinson et al. 2005; Jackson et al. 2006; Evison et al. 2012a). Here, we assess their foraging strategy when faced with a series of binary choice tests that offered colonies two food resources of varying qualities. We investigated two hypotheses: firstly, the hypothesis that a greater contrast in food quality would elicit a stronger preference for the higher quality food resource and a faster decision time when foraging. Secondly, the hypothesis that when two resources of identical quality were available, the rate at which symmetry breaking occurred would differ depending on the quality of the identical food resources. Symmetry breaking could either be stronger when food sources were of high quality due to higher ant traffic and pheromone deposition (Sumpter 2010) or could be stronger when food sources were of low quality due to a disproportionate effect of pheromone deposition on the relative trail strength (Deco et al. 2007; Perna et al. 2012; Czaczkes et al. 2015).

## Methods

### Colony set-up

Twenty colonies of *M. pharaonis* were used during the study. Each colony was initially made up of 600 workers, 20 queens and brood of various stages and was maintained at this approximate size throughout the experiments. Colonies were maintained in plastic containers (22 × 16 × 7 cm), the inner sides of which were coated with Fluon® (Whitman Plastics Ltd.) to prevent escape. Colonies were kept at 25 °C and 70 % RH and fed on 20 % sucrose solution, *ad libitum*, and three *Tenebrio molitor* larvae per week, and each was provided with an artificial nest box (45 × 32 × 12 mm) coated with foil on the sides and red acetate on the top and bottom to create a dark environment for the ants inside. The entrance hole of each nest was approximately 6 mm in diameter. Colonies were starved for 72 h prior to each trial to ensure that they were motivated to actively recruit to a food source. Sucrose solutions of 0.1, 0.5 and 1 M were made using distilled water and were coded in order to minimise observer bias when behavioural data were recorded.

### Experimental procedure

**Fig. 1:**
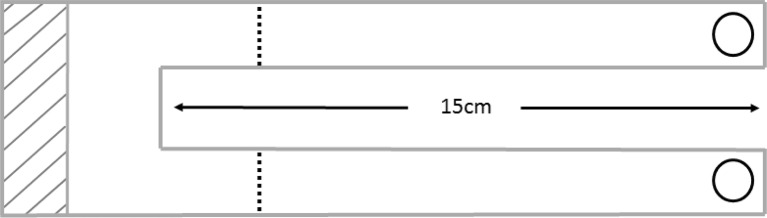
Experimental apparatus used for all bifurcation experiments. *Shaded area* indicates access area for the ants, which was ~20 cm from the nest box. Feeders are indicated by *circles* at the end of each branch. *Dashed lines* indicate the points at which ant traffic counts were made (2.5 cm from bifurcation). Each branch had a width of 15 mm

A raised Y-shaped bifurcating set-up was used to investigate colony foraging decision-making with respect to food source quality (Fig. 1). The Y-shaped bifurcation was placed into the centre of a colony’s foraging arena, and two food resources were placed at the ends of each of the two raised branches of the bifurcation. The bifurcation was accessed via a vertical stand from which the branches extended. Food was presented to the ants as 300 μl resources in 1.5 ml Eppendorf tube caps. Behavioural observations began as soon as the set-up was complete within the colony’s nest box and ants were able to access the food sources. The number of ants going to and from the food was counted at a point 2.5 cm from the branch bifurcation for 1 min every 5 min for each branch. The number of ants feeding at the feeder was also counted once every 5 min. These recordings continued for 60 min. In Experiment 1, we tested the effect of contrasting quality food sources on ant traffic: a high disparity treatment (0.1 vs. 1 M) and a low disparity treatment (0.1 vs. 0.5 M). In Experiment 2, we tested the effect of identical quality food sources on ant traffic: three food quality levels: low: 0.1 vs. 0.1 M, medium: 0.5 vs. 0.5 M and high: 1 vs. 1 M. The order of choice tests for a single colony was randomly assigned, so the possible effect of learning would not bias the results. Each colony was used once for each choice test; therefore, the number of trials per choice test was always equal to the number of colonies (20).

### Statistical analyses

All the data were analysed in R 3.1.2 (R Core Development Team 2011). In all cases, traffic on a particular branch was the sum of ants going to and from the feeder for 1 min. In Experiments 1 and 2, raw traffic counts on the two branches and the number of ants feeding at the food source were compared using a generalised linear mixed effects models (GLMM) with Poisson error distributions using the *glmmadmb* function in the *glmmADMB* package (Skaug et al. 2013). The fixed effects were time and resource quality. Paired *t* tests were carried out on the traffic counts going to each branch for each trial over 60 min. This was carried out to determine out of how many of the trials within a treatment decisions were made between the branches. The proportion of ant traffic towards the higher quality feeder (Experiment 1) or the favoured feeder (Experiment 2) in each treatment was compared for all treatments using a linear mixed effect model. The favoured feeder (Experiment 2) was the branch that had higher total traffic at the end of each 60 min experiment. When analysing this, data for the two branches were converted into a single proportion of ants visiting the favoured feeder. The first 20 min were removed so only the level of exploitation after pheromone trail establishment (rather than exploration) was compared between treatments. We used the *lmer* function from the *lme4* package to analyse these data (Bates 2007). The fixed effects were time and experimental treatment, and total foraging effort (the total number of foragers going to the feeders over the 60 min summed) was included as a covariate. Finally, we also analysed total foraging effort towards each branch using a GLMM with Poisson error distributions, with a fixed effect of experimental treatment. We controlled for non-independence of data points by including colony as a random effect in all our mixed models. Sequential Bonferroni *p* value adjustment for multiple comparisons was used for all proportion data (Sokal and Braumann 1980). Finally, to allow us to assess whether the results of our study were influenced by overcrowding within the system, we correlated ant traffic counts with counts of ants at the feeder using cor.test: poor correlations would suggest crowding at either source; strong correlations would suggest no crowding.

## Results

### Experiment 1

**Fig. 2:**
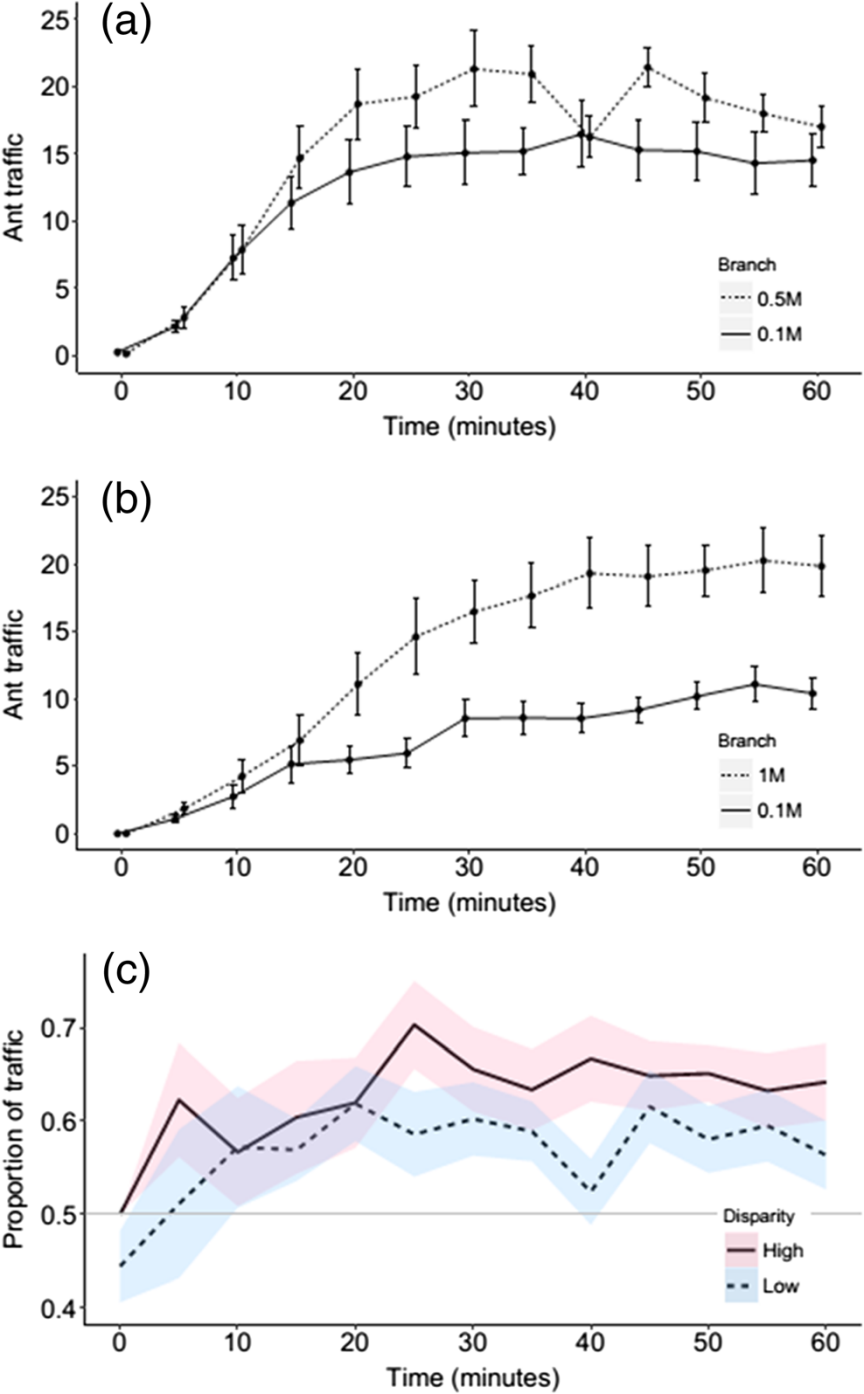
Experiment 1. **a** Mean ± s.e. number of ants travelling on a trail to/from medium quality (0.5 M) and low quality (0.1 M) food sources. This was considered to be a low contrast food quality treatment. **b** Mean ± s.e. ant foraging traffic when high quality (1 M) and low quality (0.1 M) food sources are available. This was considered to be a high contrast food quality treatment. **c** The proportion (mean ± s.e.) of ant traffic going to the high quality feeder from the low (**a**) and high (**b**) contrast treatments. The *grey horizontal line* signifies an even distribution of traffic between the two branches

Choice tests with contrasting food quality showed a significant difference in traffic between branches (Fig. 2); in both cases, the higher quality branch had greater traffic. In the low contrast treatment (0.1 vs. 0.5 M), the 0.5 M branch was favoured (glmm: *z* = 9.16, *p* < 0.001; Fig 2a). In the high contrast treatment (0.1 vs. 1 M), the 1 M branch was favoured (glmm: *z* = 22.1, *p* < 0.001; Fig 2b). The extent to which ants favoured the branch leading to the higher quality feeder in these two treatments was analysed by looking at the proportion of total ant traffic going to the higher quality feeder. The treatment with greatest disparity (0.1 vs. 1 M) was found to have a greater proportion of ants going to the higher quality feeder (lme: *z* = −2.938, *p* = 0.0035; Fig. 2c). Paired *t* tests showed that overall, the 1 M branch was favoured 12 times, the 0.1 M branch once and neither were favoured in the remaining seven trials. In the lower contrast, treatment decisions were made between the two branches less frequently; the higher quality 0.5 M branch was favoured 6 times and neither feeders were favoured in the remaining 14 trials.

### Experiment 2

**Fig. 3:**
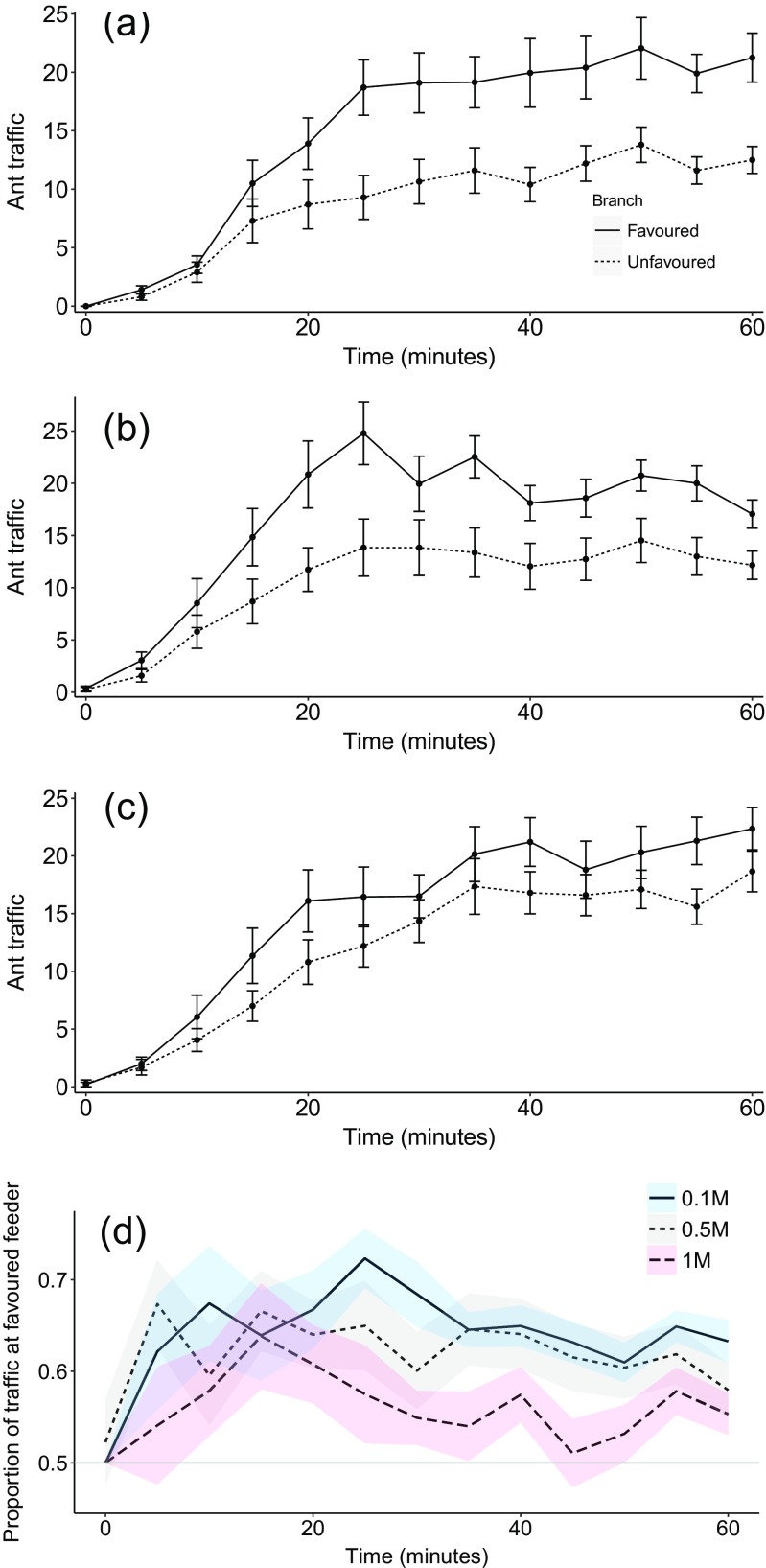
Mean ± s.e. number of ants travelling on a trail to/from equal quality feeders: **a** low quality (0.1 M), **b** medium quality (0.5 M) and **c** high quality (1 M) food sources. **d** The mean ± s.e. proportion of ant traffic on the favoured branch in each of the three identical quality treatments from Experiment 2. The *grey horizontal line* signifies an even distribution of traffic between the two branches. Each *line* corresponds to a treatment with either two low quality food sources (0.1 M), two medium quality food sources (0.5 M) or two high quality food sources (1 M)

**Fig. 4:**
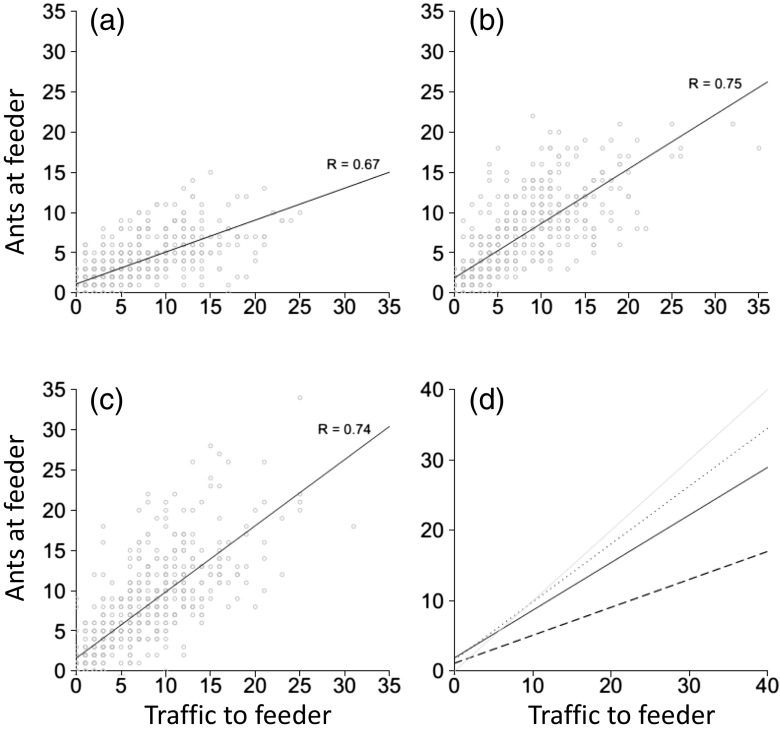
The number of ants travelling on a trail to/from a feeder (*x*-axis, Traffic) correlates strongly with the number of ants on each feeder (*y*-axis, Feeder) during the course of the experiments, whether the food quality is **a** low quality (0.1 M), **b** medium quality (0.5 M) or **c** high quality (1 M), and the slope of the relationship varies dependent on food quality **d**

In all three treatments, traffic counts showed that colonies displayed symmetry breaking to different degrees depending on food quality (Fig. 3). By analysing data using proportions, we were able to compare the extent of symmetry breaking in each treatment. The proportion of ant traffic on the favoured branch was significantly greater in the 0.1 M (low quality) treatment than in the 1 M (high quality) treatment (lme: *t* = −4.729, *p* < 0.001). It was also significantly greater in the 0.5 M (medium quality) treatment than in the 1 M treatment (*t* = −3.625, *p* < 0.001). No difference was seen between the 0.5 and 0.1 M treatments (lme: *t* = −1.014, *p* = 0.31). Paired *t* tests showed the same pattern reflected in the number of trials resulting in selection of a feeder, with 7 trials resulting in a choice in the 0.1 M treatment, 9 trials resulting in a choice in the 0.5 M treatment but only 4 trials resulted in a choice in the 1 M high quality treatment. Differences in total foraging effort (total number of ants recorded going to feeders over 60 min) were also seen between treatments (medium vs. low: glmm: *z* = 3.57, *p* = 0.0004; high vs. low: glmm: *z* = 9.72, *p* < 0.001; high vs. medium: glmm: *z* = 5.47, *p* < 0.001). However, the average ant traffic per minute for each treatment was 23.2, 26.4 and 26.5 (0.1, 0.5 and 1 M). Therefore, differences in the level of symmetry breaking were unlikely to be due to low foraging numbers in some treatments. Differences were also seen between treatments in the combined number of individuals feeding on both food sources. The high and medium quality treatments had a greater number of individuals feeding than the low quality treatment (high vs. low: glmm: *z* = 23.2, *p* < 0.001; medium vs. low: glmm: *z* = 17.8, *p* < 0.001). The number of individuals feeding at the feeders in the high quality treatment was greater than at the medium quality treatment (glmm: *z* = 6.54, *p* < 0.001). This suggests that the higher the quality of a food source, the more ants will feed at that food source. Finally, there were strong correlations between the number of ants feeding at the feeders and the ant traffic counts each of the food quality treatments (low quality: *R* = 0.67, *t*_518_ = 22.2, *p* < 0.001; medium quality: *R* = 0.75, *t*_492_ = 26.1, *p* < 0.001; high quality: *R* = 0.74, *t*_518_ = 27.4, *p* < 0.001; Fig. 4), suggesting that there was no overcrowding on either the food source or on the branches leading to the food source.

## Discussion

Here, we show that symmetry breaking at two food sources is influenced by resource quality in the Pharaoh ant. When provided with food resources of unequal quality, the branches leading to the higher quality resources were preferred (Sumpter and Beekman 2003; Beckers et al. 1993). Traffic to the higher quality feeder was greater in 45 % of trials over the two treatments and only in one trial was it higher to the lower quality feeder. The data also suggest that when there is greater disparity in quality between the resources available, the disparity in foraging traffic going to the two feeders will also increase. When provided with two food sources of equal quality, it appears that colonies were able to exploit the two resources more evenly when resources were of higher quality; symmetry breaking was more limited when both resources available were of high quality. This goes against the prediction that symmetry breaking becomes stronger with increasing food source quality (Sumpter 2010) and may be explained by Weber’s Law, i.e. that the perceived difference in pheromone concentration by the ants is important in mediating foraging flexibility (Deco et al. 2007; Perna et al. 2012; Czaczkes et al. 2015).

In Experiment 1 colonies distributed a greater proportion of their foragers towards the higher quality resource. This behaviour supports work by Sumpter and Beekman (2003) on *M. pharaonis* and is typical of this mass-recruiting species (Jackson et al. 2004; Jackson and Châline 2007; Evison et al. 2012b). The stronger allocation of workers to higher quality feeders is most likely due to a greater pheromone trail laying intensity by ants coming from the these feeders (Jackson and Châline 2007) leading to faster exploitation of the higher quality food source via positive feedback influencing the decision by nestmates to lay pheromone trail (Sumpter and Beekman 2003; von Thienen et al. 2014). A greater disparity in quality should create greater disparity in foraging effort between two food sources, a simple behaviour that is integral to colony survival (Stroeymeyt et al. 2010), and this is indeed what we found (Fig. 2). Interestingly, Experiment 2 found the rate of symmetry breaking to be food quality dependent; it was less defined when colonies were offered identical food sources that were of higher quality. In 16 out 20 trials, no differences were seen between traffic on the two branches when both branches led to 1 M sugar solution. This finding is surprising because a high resource quality has a positive effect on the number of ants feeding and the intensity of pheromone deposition, both of which should increase symmetry breaking (Sumpter 2010). We believe that this result was not caused by overcrowding on the trail or feeder as has been shown previously (Dussutour et al. 2004; Grüter et al. 2012), as we found a strong correlation between the number of ants going to the feeder and the number of ants at the feeder (ESM and Fig. 4). The correlation between ant traffic and the number of ants at the feeder never exceeded a ratio of 1:1, suggesting that ants were never waiting to feed, and that our results are not simply a result of feeder saturation and overcrowding (also see ESM), and the strength of the correlations suggests there was no crowding on the branches leading to the feeders. At an individual level, our finding is likely to be explained by the nature of pheromone deposition by individual ants. With two trails leading to high quality food sources, both trails should be strongly marked (so long as both are discovered). When two identical low quality resources are available, individuals will, in general, mark a trail very weakly or at the same level to ants that have not fed (Jackson and Châline 2007). Their trail laying intensity in this case will be more random, guided in part by the starvation level which affects the threshold at which they decide to lay pheromone, as has been shown in *L. niger* (Mailleux et al. 2006). Differences were seen between treatments in the total ant traffic; however, it is unlikely, given all treatments had on average more than 20 active foragers per minute, that this would cause the differences in distribution of ants in the three treatments.

We saw differences in the number of ants feeding; the higher the food quality, the more ants were found feeding, and this is likely to mean that more ants are laying pheromone when food was of higher quality. In support of this, previous work on ants (Josens et al. 1998) has shown that when food is of high quality, individuals will fill their crop more. Weber’s law states that the perceived difference between two stimuli may depend, largely, on the proportional size of the two stimuli (Deco et al. 2007; Perna et al. 2012; Czaczkes et al. 2015), and there is increasing evidence that the response of individual ants to pheromone trails of different strengths follows this pattern (Perna et al. 2012; von Thienen et al. 2014). This will likely mean that individuals feeding on high quality food will lay more pheromone and, if trail choice follows Weber’s Law, it may be a mechanism by which a colony could more efficiently exploit multiple high quality food resources. Indeed, our results are consistent with trail choice following by Weber’s Law. When pheromone deposition intensity on the trail network is generally low, pheromone deposition decisions of individual ants will have a disproportionate effect on the relative trail strength and promote symmetry breaking, whereas when food quality is high, the proportional difference in pheromone deposition may be smaller because a large proportion of ants will deposit pheromone on both branches. This may be the reason why we see weaker symmetry breaking in the high quality treatment compared to the low quality treatment. The mechanism underlying this phenomenon requires further investigation but could be mediated by behaviours such as u-turning while trail laying (Hart and Jackson 2006; Evison et al. 2008; Jackson et al., 2011). This is performed by a small proportion of the foraging population and allows individuals to make a disproportionately large contribution to the pheromone trail, which increases the chances of a trail being followed even if it is competing with a trail that was discovered first (Reid et al. 2012).

Our result does not follow the symmetry breaking behaviour predicted by the model of Sumpter and Beekman (2003); rather, the effect of trail laying intensity in conjunction with Weber’s law is a possible explanation for what we have found in this study. When resources in an environment are of high quality, extreme symmetry breaking could be costly as it forces colonies to focus on one food source. The ability to limit asymmetrical foraging when other resources in the area are equally profitable would increase foraging flexibility and ultimately colony fitness. In a natural environment, strong symmetry breaking might be prevented by the presence of negative feedback mechanisms, such as crowding on the trail and at food sources (Grüter et al. 2012; Czaczkes et al. 2013), allowing colonies to exploit multiple food sources more equally and efficiently, and make foraging more robust to changing conditions (Dussutour et al. 2004; Czaczkes et al. 2013). Pheromone concentrations are likely to fall within a wide range of concentrations depending on the current environment, as well as being influenced by the size of the colony involved in forming trails. By following Weber’s law, concentration ratios remain stable despite highly variable underlying pheromone concentrations (von Thienen et al. 2014). Together, the results of this study give further evidence that mass recruitment may be more flexible than was previously considered (Beckers et al. 1990; Detrain and Deneubourg 2008; Dussutour et al., 2009).

## Electronic supplementary material

Below is the link to the electronic supplementary material.

ESM 1 (DOCX 381 kb)
