# Supplementary material for: Symmetry breaking in mass-recruiting ants: extent of foraging biases depends on resource quality
Source: Behav Ecol Sociobiol. 2016 Jul 30;70(11):1813–20. doi: 10.1007/s00265-016-2187-y (PMC5054046; doi:10.1007/s00265-016-2187-y)
Supplement: Supplementary file 1 — (DOCX 381 kb) [file 265_2016_2187_MOESM1_ESM.docx]

**Electronic Supplementary material**

**Symmetry breaking in mass-recruiting ants: extent of foraging biases depends on resource quality**

R. I’Anson Price^1,2^, C. Grüter^2^, W. O. H Hughes^3^ and S. E. F. Evison^4*^

1. School of Biology, University of Leeds, Leeds, UK

2. Department of Ecology and Evolution, Biophore, University of Lausanne, 1015 Lausanne, Switzerland

3. School of Life Sciences, University of Sussex, Brighton, BN1 9QG, UK

4. Department **of Animal and Plant Sciences, University of Sheffield, S10 2TN, UK**

*Corresponding author: S. E. F. Evison

Email: s.evison@sheffield.ac.uk

Tel: +44 114 222 4372

**Figure S1.**

The ‘feeder’ is an upturned 1.5ml Eppendorf cap and contains 300µl of sucrose solution. When the feeder is provided to a large Pharaoh’s ant colony (~3000 individuals), the area and volume available to feed from allows 30-40 individuals to access food. This is consistently higher than the number of ants found at the feeders during experiments with our smaller colonies of ~600 individuals, suggesting that there was no problem of overcrowding using this feeding system.


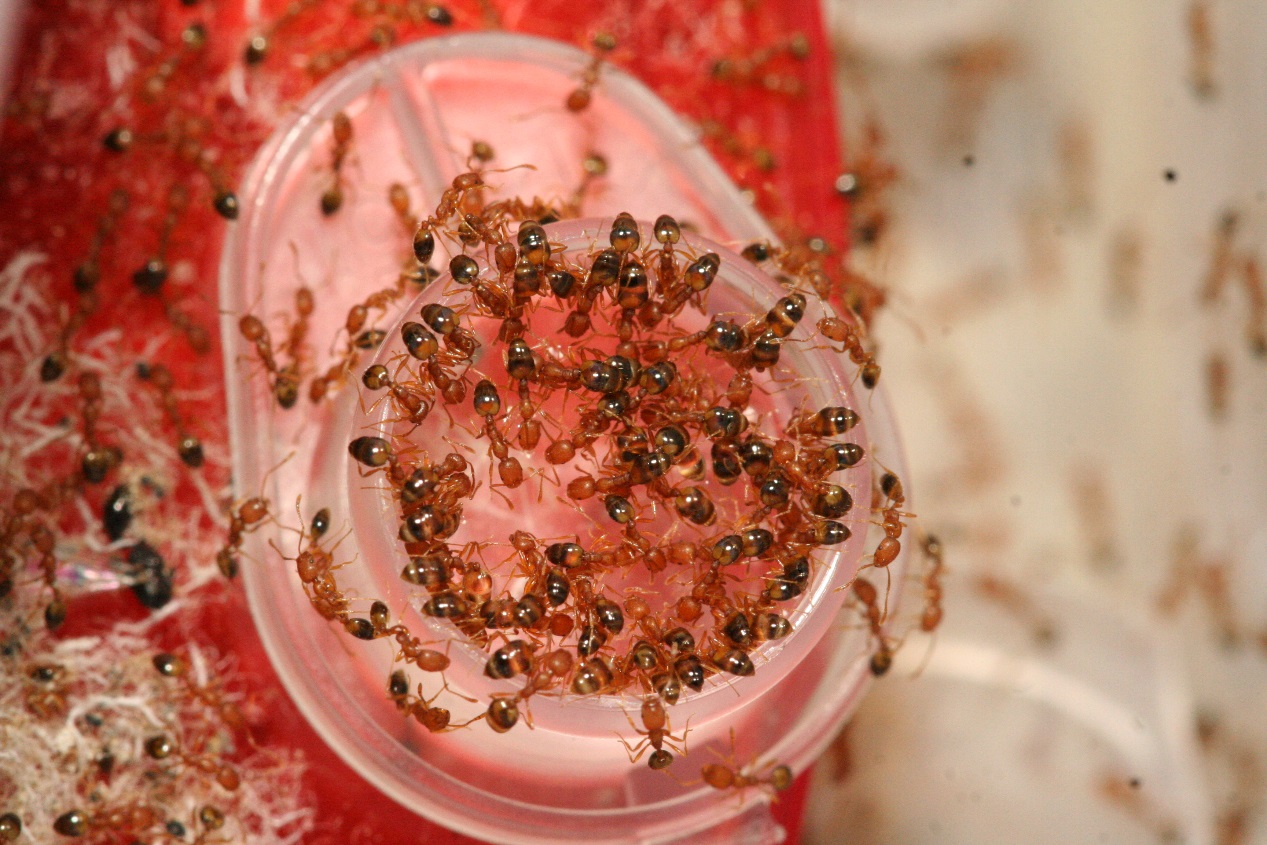


The percentage of times that the number of individuals at the feeders fell within the estimated feeder saturation range of 30-40 was extremely low, and never above the higher boundary of this estimate in both experiments:

Experiment 1. 0.45% (occurring at 25, 35, 40, 45 and 55 minutes)

Experiment 2. 0.06% (occurring only once at 15 minutes)

The percentage of times that the number of individuals going to the feeder over 1 minute (traffic counts) fell within the estimated feeder saturation range of 30-40 was also extremely low, and never above the higher boundary of this estimate in both experiments:

Experiment 1. 0%

Experiment 2. 0.2% (occurring twice at 20 minutes and once at 40 minutes)

**Figure S2.** The number of ants feeding at each feeder during the course of the experiment 2. **
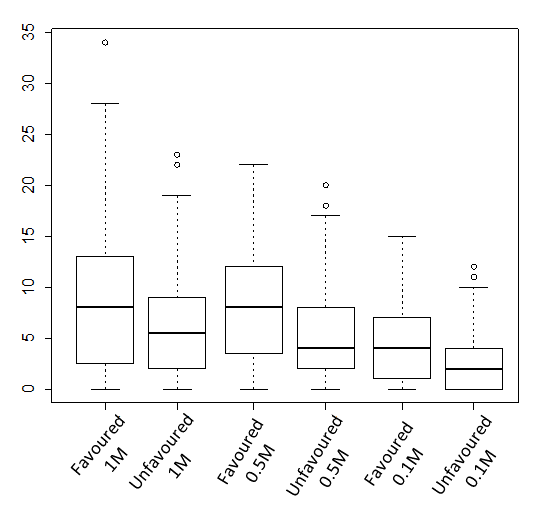
**

The only point at which the number of ants feeding at the feeder fell within our estimated feeder saturation range of 30-40 is a single incidence found in the high quality treatment at the favoured feeder.
